# Association of admission cortisol levels with outcomes and treatment response in patients at nutritional risk: A secondary analysis of a randomized clinical trial

**DOI:** 10.1186/s12937-023-00881-6

**Published:** 2023-11-15

**Authors:** Mirsada Durmisi, Nina Kaegi-Braun, Natasha A. Müller, Carla Wunderle, Pascal Tribolet, Zeno Stanga, Beat Mueller, Philipp Schuetz

**Affiliations:** 1 Medical University Department, Division of General Internal and Emergency Medicine, Kantonsspital Aarau, Tellstrasse 25, Aarau, CH-5001 Switzerland; 2 Medical Faculty, University of Basel, Basel, Switzerland; 3 Department of Health Professions, Bern University of Applied Sciences, Bern, Switzerland; 4 Faculty of Life Sciences, University of Vienna, Vienna, Austria; 5 Division of Diabetes, Endocrinology, Nutritional Medicine, and Metabolism, Inselspital Bern, Bern University Hospital, University of Bern, Bern, Switzerland

**Keywords:** Cortisol, HPA-axis, Critical Illness, Acute and chronic stress, Mortality, Nutritional risk, Nutritional support

## Abstract

**Introduction:**

Cortisol is a metabolically active stress hormone that may play a role in the pathogenesis of malnutrition. We studied the association between admission cortisol levels and nutritional parameters, disease severity, and response to nutritional support among medical inpatients at nutritional risk.

**Methods:**

Admission cortisol was measured in a subset of 764 patients participating in the Effect of Early Nutritional Support on Frailty, Functional Outcomes, and Recovery of Malnourished Medical Inpatients Trial (EFFORT), a multicentre, randomized-controlled trial that compared individualized nutritional support with usual nutritional care.

**Results:**

Overall, mean cortisol levels were 570 (± 293) nmol/L and significantly higher in patients with high nutritional risk (NRS ≥ 5) and in patients reporting loss of appetite. Cortisol levels in the highest quartile (> 723 nmol/l) were associated with adverse outcomes including mortality at 30 days and 5 years (adjusted HR 2.31, [95%CI 1.47 to 3.62], p = 0.001 and 1.51, [95%CI 1.23 to 1.87], p < 0.001). Nutritional treatment tended to be more effective regarding mortality reduction in patients with high vs. low cortisol levels (adjusted OR of nutritional support 0.54, [95%CI 0.24 to 1.24] vs. OR 1.11, [95%CI 0.6 to 2.04], p for interaction = 0.134). This effect was most pronounced in the subgroup of patients with severe malnutrition (NRS 2002 ≥ 5, p for interaction = 0.047).

**Conclusion:**

This secondary analysis of a randomized nutritional trial suggests that cortisol levels are linked to nutritional and clinical outcome among multimorbid medical patients at nutritional risk and may help to improve risk assessment, as well as response to nutritional treatment.

**Trial Registration:**

ClinicalTrials.gov Identifier: NCT02517476.

**Supplementary Information:**

The online version contains supplementary material available at 10.1186/s12937-023-00881-6.

## Introduction

Disease-related malnutrition is a complex syndrome resulting from different mechanisms including inadequate intake of food and starvation, acute and chronic disease including polypharmacy, disease-related inflammatory mechanisms and compromised assimilation of nutrients, as well as immobility, advanced ageing and social isolation [1]. Recently, disease-related inflammation has been found to be key contributing factor, directly influencing anorexia with reduced intake of energy and protein. Cytokines have been shown to affect brain circuits that control food intake, delay gastric emptying, and influence skeletal muscle catabolism. In addition, endocrine changes may also contribute significantly to the pathogenesis of malnutrition [2]. Particularly, certain endocrine changes result in response to illness resulting in catabolism including an increase in cortisol concentrations, a down-regulation of sex hormones and peripheral growth hormone resistance. These changes again may occur in response to inflammation with cytokines modulating the hypothalamic-pituitary-adrenal axis response at each level and stimulate the release of stress hormones - including cortisol [3, 4]. Cortisol is a metabolically active hormone that has both, catabolic and anabolic effects. It acts catabolically through lipolysis of peripheral adipose tissue and proteolysis of skin, muscle, and lymphatic tissue. The anabolic effects of cortisol including gluconeogenesis and glycogen synthesis in the liver ensure that energy obtained from catabolism is made available to the body as needed. In stressful situations - or during prolonged food deprivation such as in acutely-ill patients - these processes are activated in order to provide the body with energy [5]. Any form of illness acts as a stressor and increases energy demand. However, patients may react differently to stress or illness depending on the severity and duration. While chronic stress activates the hypothalamic-pituitary-adrenal (HPA) axis and directly stimulates cortisol production, acute stress triggers the sympathetic-adrenal medulla and the release of primarily catecholamines [6]. Under normal conditions cortisol (as part of the HPA axis) is produced in a specific diurnal rhythm, including a peak in the morning and a trough at midnight, with small pulsatile fluctuations in between. However, acute illness causes disruptions such as with the nadir at night. Also, chronic disease can disrupt pulsatile and circadian cortisol production, which leads to permanently elevated cortisol [4, 7].

With its catabolic activities, cortisol may thus play a critical role in the pathogenesis of malnutrition in the acutely-ill medical patient [8, 9]. Still, while several studies have investigated the significance of cortisol in the critically ill patients [10–15], there is a lack of research investigating the potential role of cortisol in the pathophysiology of malnutrition outside critical care. Such knowledge may help to better characterise the malnourished patient regarding nutritional risk and response to nutritional treatment. Herein, using data of a recent large-scale randomized controlled nutritional trial (EFFORT) [16], we studied the association of admission cortisol levels with nutritional and clinical outcomes, as well as treatment response among medical inpatients at nutritional risk.

## Material & methods

### Study design and setting

This is a secondary analysis of the Effect of Early Nutritional Support on Frailty, Functional Outcomes, and Recovery of Malnourished Medical Inpatients Trial (EFFORT), a randomized-controlled trial conducted in eight Swiss hospitals between April 2014 and February 2018 [16]. In the original trial, patients were randomly assigned (1:1) within 48 h after hospital admission to receive either individualized nutritional support (intervention group) or usual hospital food (control group). For intervention group patients, energy and protein goals were defined for each participant by a well-informed and registered dietitian who then developed individualized oral nutrition plans. If nutritional goals were not achieved (> 75%) within 5 days via oral feeding and nutritional supplements, escalation to enteral tube feeding and parenteral nutrition was proposed. Control group participants received usual hospital food without additional nutritional counselling. Follow-up phone interviews were performed by blinded study nurses at 30 and 180 days after inclusion in the trial. Family members and/or family physicians were contacted if confirmation of survival status was necessary. Detailed information regarding study design and treatment algorithms have been published previously [16].

The Ethics Committee of North-western Switzerland (EKNZ; 2014_001) approved the study protocol, and all participants or their authorized representatives provided written informed consent. The study was conducted in accordance with the principles of the Declaration of Helsinki. The trial was registered retrospectivel at ClinicalTrials.gov in August 2015 (https://clinicaltrials.gov/ct2/show/NCT02517476).

### Patient population

The original (EFFORT) trial included all inpatients (≥ 18 years) with an expected hospital stay of more than four days, a Nutritional Risk Screening 2002 (NRS 2002) total score of 3 points or higher, and willingness to provide informed consent. The NRS 2002 is a validated tool covering nutritional status (BMI, weight loss, reduction of food intake), disease severity, and age. Exclusion criteria were: initial admission to a surgical unit or intensive care (ICU); inability to ingest nourishment orally or tolerate nutritional support at time of admission, nutritional support prior to admission, and any individuals with contraindications for nutritional support. In the original trial, other patients with certain diseases or conditions (ex. acute pancreatitis, acute liver failure or cystic fibrosis, anorexia nervosa, stem cell transplantation, terminal status, and history of gastric bypass surgery) were excluded. For this secondary analysis, we only included patients from the main study centre (Kantonsspital Aarau) where cortisol was measured as part of an ancillary project.

### Cortisol assessment and classification

During the original trial, blood was systematically collected upon inclusion to the study and stored for further batch analyses. Samples were taken in the morning (i.e. 6–7 am) within 48 h of admission. We stratified admission cortisol levels into quartiles and defined two categories: patients with levels in the highest quartile (> 723 nmol/l) were defined as “high cortisol” while patients in the lower 3 quartiles (≤ 723 nmol/l) were assigned the “low cortisol” group.

### Outcomes

Our primary endpoint was all-cause mortality within 5 years (for prognostic analyses) and within 30 days (for the evaluation of treatment response according to the initial trial). Short- and long-term secondary endpoints were predefined as: adverse clinical outcome within 30 days (including all-cause mortality; admission to ICU from a medical ward; non-elective hospital readmission after discharge; novel major complications - including adjudicated nosocomial infection, respiratory failure, a major cardiovascular event (i.e., stroke, intracranial bleeding, cardiac arrest, myocardial infarction or pulmonary embolism); acute renal failure; gastrointestinal failure (i.e., haemorrhage, intestinal perforation, acute pancreatitis); length of hospital stay (LOS); loss of function (10% decrease in Barthel Index, performance of daily living activities on a scale of 0–100, with higher scores indicating fewer problems with self-care and mobility); mean protein and energy intake within 10 days; and finally, handgrip strength (HGS).

### Statistical analysis

Continuous variables were expressed as mean and standard deviation (SD), and binary and categorial variables as counts and percentages. Pearson’s χ^2^ test was used to compare frequencies and Student’s t-test for continuous variables. First, univariate and multivariate linear regression analysis were used to identify predictors of high cortisol concentrations. We tested for multicollinearity calculating variance inflation factors (VIF); mean VIF was 1.67 and therefore we assume low collinearity between the independent variables. The final multivariate model includes demographics (age, gender), NRS overall score, main diagnosis and different laboratory parameters.

Second, we investigated the prognostic value of cortisol and outcomes. We used Cox regression models to analyse mortality and reported hazard ratios (HR) with 95% confidence intervals (95% CI). Kaplan Meier Curves were implemented for graphical display. Linear and logistic regression were calculated for all other prognostic endpoints; reporting odds ratio (OR) or coefficient (Coeff) with 95% CI. We adjusted for predefined covariates including sex, age, baseline nutritional risk (based on NRS 2002), main diagnosis (i.e., infection, metabolic diseases), comorbidities (i.e., cancer, renal failure, diabetes, chronic obstructive pulmonary disease) (Model 1), as well as C reactive protein (CRP) (Model 2).

Third, to explore the predictive value of cortisol regarding response to nutritional support, we calculated the association between individual nutritional support and clinical outcomes and stratified the cohort according to cortisol levels as mentioned above. To additionally explore for effects regarding inflammation and cortisol in a subgroup analysis, we divided patients into two groups according to their admission CRP level, degree of malnutrition risk, and age. Based on results from a former analysis [17], low CRP was defined as < 100 mg/l and high CRP as ≥ 100 mg/l. Based on admission NRS 2002 score, we categorized patients as “moderately malnourished” (NRS 2002 score 3 and 4 points), or “severely malnourished” (NRS 2002 score ≥ 5 points). Finally, the cohort was stratified by age (< 75 and ≥ 75 years). For this subgroup, we also calculated an interaction analysis. Due to multiple testing (7 analyses), we consider p values of < 0.007 to be statistically significant. All statistical analyses were performed with STATA 15.1 (Stata Corp, College Station, TX, USA). A P value < 0.05 (for a two-sided test) was considered statistically significant.

## Results

### Patient population – baseline characteristics

**Fig. 1:**
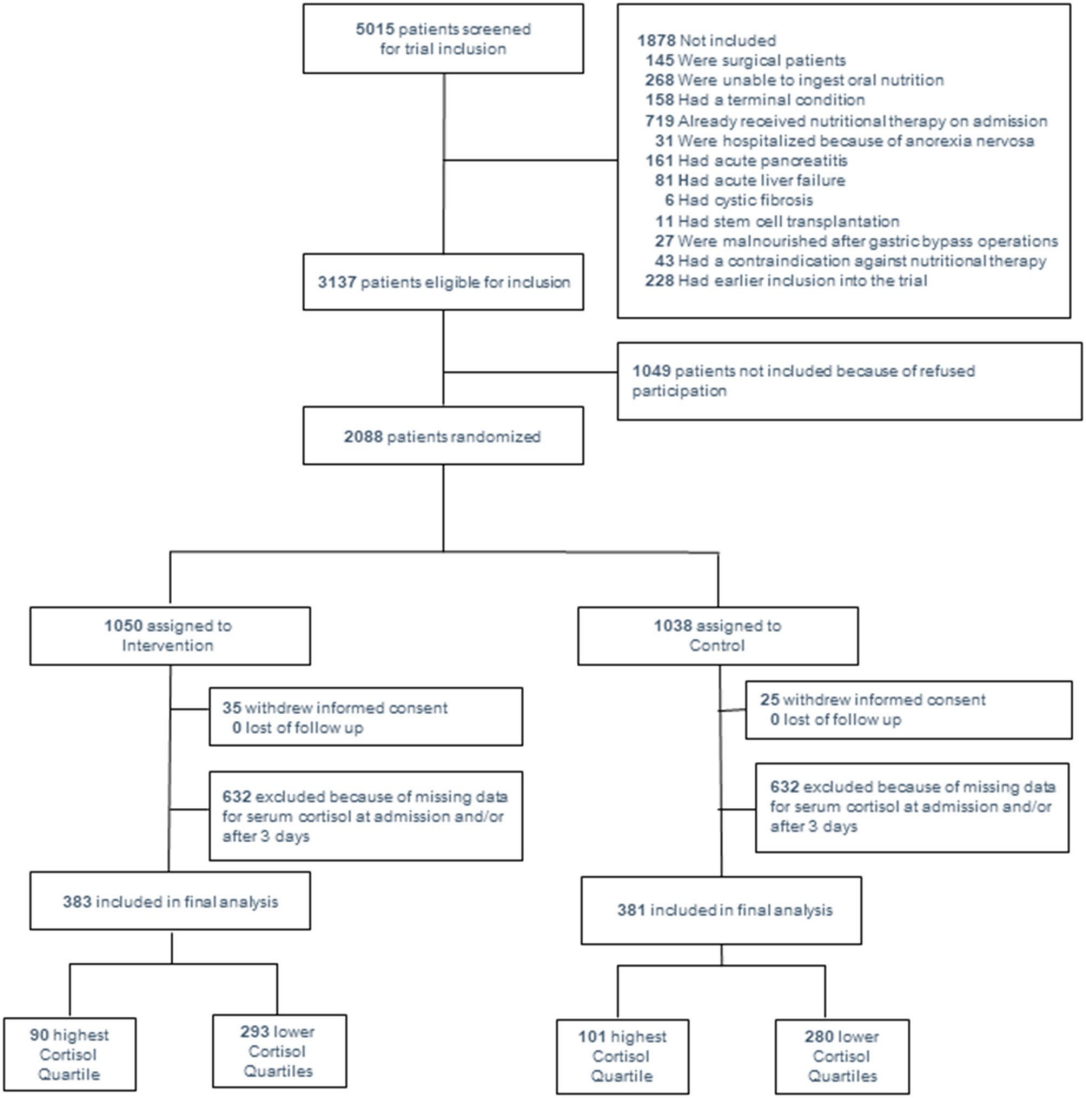
Study Flow Abbrevations: IC, informed consent Reasons for exclusion: 145 surgigal patients, 268 unable to ingest oral nutrition, 158 terminal condition, 719 already receiving nutritional therapy upon admission, 31 anorexia nervosa, 161 acute pancreatitis, 81 acute liver failure, 6 cystic fibrosis, 11 stem-cell transplantation, 27 malnutrition after gastric bypass operation, 43 contraindication against nutritional support, 228 earlier inclusion in the trial

For this secondary analysis we included 764 patients with available admission cortisol levels from one study centre out of the 2028 patients from the original trial. A total of 90 of 383 patients in the intervention group and 101 of 381 from the control group were classified as “high cortisol” (cortisol levels > 723 nmol/l) (p = 0.337) (Fig. 1). Table 1 summarizes study population baseline characteristics, stratified by high vs. low cortisol level. Mean cortisol levels were 446 (± 182) nmol/l in the low and 942 (± 245) nmol/l in the high cortisol group, respectively. Median age was 73 years, 54% were male. In the high cortisol group, patients were more severely malnourished (p = 0.021) and infectious disease diagnoses upon admission were more prevalent (35% vs. 25%, p = 0.013). There was no significant difference in the frequency of other admission diagnosis or comorbidities (except for chronic obstructive pulmonary disease which was more frequent in the low cortisol group). CRP (111 mg/l vs. 66 mg/l, p < 0.001) and creatinine (152 µmol/l vs. 122 µmol/l, p = 0.01) levels were also more elevated in the high cortisol group, while mean albumin level (28 g/l vs. 26 g/l, p < 0.001) was lower.

**Table 1:** Baseline characteristics overall and stratified by low cortisol and high cortisol at admission

	Overall	Low cortisol^3^	High cortisol^4^	p-value
n	764	573	191	
**Sociodemographics**				
Age years, mean (SD)	73 (13)	73 (13)	75 (12)	0.16
Male sex	413 (54%)	317 (55%)	96 (50%)	0.22
**Nutritional assessment**				
BMI kg/m2, mean (SD)	25 (5)	25 (5)	25 (5)	0.68
Weight at admission kg, mean (SD)	71 (16)	72 (16)	72 (16)	0.84
Height cm, mean (SD)	168 (9)	168 (9)	167 (9)	0.3
**NRS 2002 score (NRS 2002)**				
NRS 2002 score = 3	206 (27%)	169 (30%)	37 (19%)	**0.021**
NRS 2002 score = 4	300 (39%)	220 (38%)	80 (42%)	
NRS 2002 score > 5	258 (34%)	184 (32%)	74 (39%)	
**Steroids intake**				
Oral steroids	101 (14%)	88 (16%)	13 (7%)	**0.002**
Inhaled steroids	211 (28%)	68 (12%)	17 (9%)	0.26
**Admission diagnosis**				
Infection	211 (28%)	145 (25%)	66 (35%)	**0.013**
Cancer	181 (24%)	131 (23%)	50 (26%)	0.35
Cardiovascular disease	86 (11%)	68 (12%)	18 (9%)	0.35
Failure to thrive	54 (7%)	42 (7%)	12 (6%)	0.62
Lung disease	45 (6%)	41 (7%)	4 (2%)	**0.01**
Gastrointestinal disease	56 (7%)	44 (8%)	12 (6%)	0.52
Metabolic disease^1^	30 (4%)	21 (4%)	9 (5%)	0.52
**Comorbidities**				
Hypertension	460 (60%)	347 (61%)	113 (59%)	0.73
Malignant disease	284 (37%)	209 (37%)	75 (39%)	0.49
Chronic kidney disease	280 (36%)	202 (35%)	78 (41%)	0.17
Coronary heart disease	181 (24%)	141 (25%)	40 (21%)	0.3
Diabetes mellitus^2^	178 (23%)	127 (22%)	51 (27%)	0.2
Congestive heart failure	139 (18%)	107 (19%)	32 (17%)	0.55
Chronic obstructive pulmonary disease	101 (13%)	84 (15%)	17 (9%)	**0.042**
Peripheral arterial disease	79 (10%)	61 (11%)	18 (9%)	0.63
Stroke	71 (9%)	51 (9%)	20 (10%)	0.52
**Laboratory at admission**				
Cortisol nmol/l, mean (SD)	570 (293)	446 (182)	942 (245)	**< 0.001**
CRP mg/l, mean (SD)	77 (80)	66 (72)	111 (94)	**< 0.001**
Creatinine umol/l, mean (SD)	130 (138)	122 (119)	152 (180)	**0.01**
GFR ml/min, mean (SD)	35 (16)	36 (16)	33 (15)	0.066
Albumin g/l, mean (SD)	27 (6)	28 (6)	26 (5)	**< 0.001**
Phosphat mmol/l, mean (SD)	1.0 (0.4)	1.0 (0.3)	0.9 (0.4)	0.95

The two-sample-t-test was used to compare the baseline characteristics between the intervention and the control group for the continuous variables and Pearson’s Chi-squared-test for binary and categorical variables. Data are expressed as number (%) unless otherwise indicated

Abbrevations: SD, standard deviation; BMI, body mass index; NRS, nutrional risk score; kg, kilogram; CRP, C-reactive protein; GFR, glomerular filtration rate

^1^ Metabolic disease included, but was not limited to, ketoacidosis, hypo- and hyperglycemia and electrolyte disturbances

including hypo- and hypernatriaemia, as well as hypo- and hyperkaliemia

^2^ Type 1 or type 2

^3^ Low cortisol = ≤ 723 nmol/l

^4^ High cortisol = > 723 nmol/l

### Association between cortisol levels and patient characteristics

The association between different baseline characteristics and admission cortisol levels was studied using univariate and multivariate regression models (Table 2). Age was a significant predictor for elevated cortisol levels. Higher nutritional risk assessed by NRS 2002 was also associated with higher cortisol levels. To further investigate this relationship, we calculated individual NRS 2002 score components and found higher cortisol levels in patients with 25-50% food intake compared to those with > 75% normal intake in the week prior to hospitalization. There was also an association between loss of appetite and higher cortisol levels. Patients hospitalized for infectious diseases showed higher cortisol levels, while those with lung disease had lower results at admission. In the multivariate calculations, results remained robust for age (difference per year of 2 mmol/l, [95% CI 0 to 4], p = 0.016), NRS score ≥ 5 points (difference pre point of 61 mmol/l, [95% CI 6 to 117], p = 0.031), CRP (difference per 10 unit increase of 13 mmol/l, [95% CI 10 to 17], p = < 0.001), creatinine (difference per unit increase of 3 mmol/l, [95%CI 1 to 5], p = 0.015), and a main diagnosis of lung disease (difference − 109 mmol/l, [95% CI [-211 to -7], p = 0.036). Additional results are shown in the supplemental Table 1.

**Table 2:** Association of different baseline characteristics with cortisol level at time of admission

		Univariate		Multivariate *	
	Cortisol (nmol/l), mean (SD)	Coefficient (95% CI)	p-value	Coefficient (95% CI)	p-value
**Sociodemographics**					
**Female**	573 (301)	reference			
**Male**	568 (286)	-5 (-47 to 37)	0.811	-17 (-59 to 25)	0.431
**Age**		2 (1 to 4)	**0.006**	2 (0 to 4)	**0.016**
**Anthropometric measurements**					
**Height, cm**		-4 (1 to 4)	0.174		
**Nutritional status**					
**NRS 2002 score (NRS 2002)**					
NRS 2002 score = 3	517 (286)	reference		reference	
NRS 2002 score = 4	571 (275)	54 (3 to 106)	**0.04**	31 (-21 to 83)	0.242
NRS 2002 score = ≥ 5	611 (313)	94 (41 to 147)	**0.001**	61 (6 to 117)	**0.031**
**Nutritional intake** ^**1**^					
75–100%	507 (248)	reference			
50–75%	550 (303)	42 (-38 to 123)	0.301		
25–50%	597 (293)	90 (13 to 167)	**0.022**		
0–25%	567 (294)	60 (-24 to 144)	0.163		
**Loss of appetite**					
No	507 (266)	reference			
Yes	578 (296)	71 (5 to 137)	**< 0.001**		
**Main diagnosis**					
**Cancer**	561 (323)	-12 (-61 to 37)	0.638	52 (-17 to 121)	0.14
**Cardiovascular disease**	611 (270)	46 (-20 to 112)	0.168	138 (53 to 222)	**0.001**
**Infection**	633 (293)	86 (40 to 133)	**< 0.001**	22 (-48 to 93)	0.533
**Renal disease**	625 (258)	57 (-34 to 149)	0.218	95 (-31 to 221)	0.139
**Frailty**	562 (240)	-9 (-90 to 72)	0.831	46 (-56 to 148)	0.377
**Lung disease**	369 (273)	-214 (-301 to -127)	**< 0.001**	-109 (-211 to -7)	**0.036**
**Metabolic disease** ^**2**^	632 (338)	64 (-43 to 172)	0.239	149 (29 to 269)	**0.015**
**Comorbidities**					
**Hypertension**	579 (299)	21 (-22 to 63)	0.337		
**Malignant disease**	584 (313)	21 (-22 to 64)	0.335		
**Chronic renal disease**	590 (257)	32 (-12 to 75)	0.152		
**Chronic heart failure**	586 (271)	20 (-34 to 74)	0.474		
**Diabetes mellitus** ^**3**^	584 (290)	18 (-32 to 67)	0.48		
**Chronic obstructive pulmonary disease**	487 (323)	-96 (-157 to -35)	**0.002**		
**CHD**	573 (263)	4 (-45 to 53)	0.876		
**Labarotory parameters**					
**CRP (per 10 mg/l)**		11 (8 to 13)	**< 0.001**	13 (10 to 17)	**< 0.001**
**Albumin (per 10 g/l)**		-72 (-110 to -35)	**< 0.001**	43 (-6 to 91)	0.084
**Creatinine (per 10 umol/l)**		2 (1 to 4)	**0.005**	3 (1 to 5)	**0.015**
**GFR ml/min**		-2 (-3 to 0)	0.086		
**Phosphat mmol/l**		-11 (-73 to 51)	0.731	-74 (-154 to 5)	0.067

Univariate and multivariate linear regression analysis to identify predictors of high cortisol concentrations upon admission. Values are mean (SD), and regression coefficients (95% CI) in nmol/l. Coefficients indicate the decrease or increase of cortisol concentrations in patients presenting with the characterstic compared to patients without the characteristic

Abbrevations: SD, standard deviation; cm, centimeter; BMI, body mass index; NRS, nutrional risk score; kg, kilogram; CHD, coronary heart disease;

CRP, C-reactive protein; GFR, glomerular filtration rate

^1^ Nutritional intake in percent of the prior normal nutritional intake

^2^ Metabolic disease included, but was not limited to, ketoacidosis, hypo- and hyperglycemia and electrolyte disturbances

including hypo- and hypernatriaemia, as well as hypo- and hyperkaliemia

^3^ Type 1 or Type 2

### Association between high cortisol levels and clinical outcomes

Mortality after 30 days, 180 days and 5 years was 10.7%, 28.8% and 59.4%, respectively. Table 3 shows associations between cortisol levels and clinical outcomes. In patients with high cortisol levels, mortality risk within the first 30 days was doubled compared to those with lower cortisol levels (18% vs. 8%, adjusted HR 2.31, [95% CI 1.47 to 3.62], p < 0.001). Similar results were found for 180-day mortality (adjusted HR 1.83, [95% CI 1.38 to 2.43], p < 0.001) and 5-year mortality (adjusted HR 1.51, [95% CI 1.23 to 1.87], p = 0.001).

**Table 3:** Prognostic value of admission serum cortisol levels on mortality rates and other secondary clinical and nutritional outcomes

		Adjusted model 1 *		Adjusted model 2 **	
*Primary outcome*	p-value	HR (95% CI)	p-value	HR (95% CI)	p-value
30-day mortalitiy					
Low cortisol^3^		reference		reference	
High cortisol^4^	**< 0.001**	2.31 (1.47 to 3.62)	**< 0.001**	1.89 (1.18 to 3.02)	**0.008**
***Secondary long-term outcomes***					
180-day mortality					
Low cortisol^3^		reference		reference	
High cortisol^4^	**< 0.001**	1.83 (1.38 to 2.43)	**< 0.001**	1.68 (1.26 to 2.25)	**< 0.001**
5-year mortality					
Low cortisol^3^		reference		reference	
High cortisol^4^	**0.001**	1.51 (1.23 to 1.87)	**< 0.001**	1.45 (1.17 to 1.8)	**0.001**
***Secondary short-term outcomes (30 days)***	p-value	**OR (95% CI)**	**p-value**	**OR (95% CI)**	**p-value**
Adverse clinical outcomes					
Low cortisol^3^		reference		reference	
High cortisol^4^	**0.031**	1.54 (1.06 to 2.23)	**0.023**	1.45 (0.99 to 2.13)	0.056
Loss of function^1^					
Low cortisol^3^		reference		reference	
High cortisol^4^	0.104	1.46 (0.93 to 2.3)	0.104	1.29 (0.81 to 2.08)	0.286
	**p-value**	**Coefficient (95% CI)**	**p-value**	**Coefficient (95% CI)**	**p-value**
Length of hospital stay					
Low cortisol^3^		reference		reference	
High cortisol^4^	**< 0.001**	2 (1 to 3)	**< 0.001**	2 (1 to 3)	**0.001**
Mean protein intake per day, g/d^2^					
Low cortisol^3^		reference		reference	
High cortisol^4^	**0.005**	-5 (-9 to -1)	**0.011**	-4 (-8 to 0)	**0.044**
Mean caloric intake per day, kcal/d^2^					
Low cortisol^3^		reference		reference	
High cortisol^4^	**0.005**	-126 (-229 to -23)	**0.017**	-91 (-196 to 14)	**0.09**
Change in handgrip strength (HGS), kg					
Low cortisol^3^		reference		reference	
High cortisol^4^	**0.032**	1 (0 to 3)	0.081	1 (0 to 3)	0.107

Multivariable logistic regression models reporting hazard or odds ratios according to cortisol concentrations. Continous variables were assessed through linear regression models, results are expressed as coefficients

Abbreviations: n, number: SD, standard deviation; 95% CI, 95% confidence interval; HR, hazard ratio; OR, odds ratio; kg, kilograms; CRP, C-reactive protein; kcal/d, calories per day; g/d, grams per day

* adjusted for age, sex, NRS, main diagnosis, comorbidities and trial intervention

** adjusted for age, sex, NRS, main diagnosis, comorbidities, trial intervention and CRP

^1^ Loss of function definded as 10% decrease in Barthel index

^2^ until day 10 of hospitalisation

^3^ Low cortisol: ≤ 723 nmol/l

^4^ High cortisol: > 723 nmol/l

Several secondary short-term outcomes also showed an association with high cortisol levels. These included: adverse clinical outcome (OR 1.54, [95% CI 1.06 to 2.23], p = 0.023), length of stay (difference of 2.16 days, [95% CI 1.05 to 3.26], p < 0.001), mean protein intake per day (until day 10 of hospitalization) (difference − 5 g, [95% CI -9 to -1], p = < 0.011), and mean caloric intake per day (difference − 126 calories, [95% CI -229 to -23], p = 0.017). The majority of results remained robust after additional adjusting for admission CRP levels (Supplemental Table 2).

### Association of high cortisol and clinical outcomes, stratified by admission CRP levels

To better understand the influence of inflammation and cortisol levels, a subgroup analysis stratified by high CRP (≥ 100 mg/l) and low CRP (< 100 mg/l) admission levels was performed (Supplementary Table 2). Most results remained unchanged. However, increases in short- and long-term risk of mortality was more pronounced in the high CRP group, while reduction of caloric and protein intake was more pronounced in the low CRP group.

**Fig. 2:**
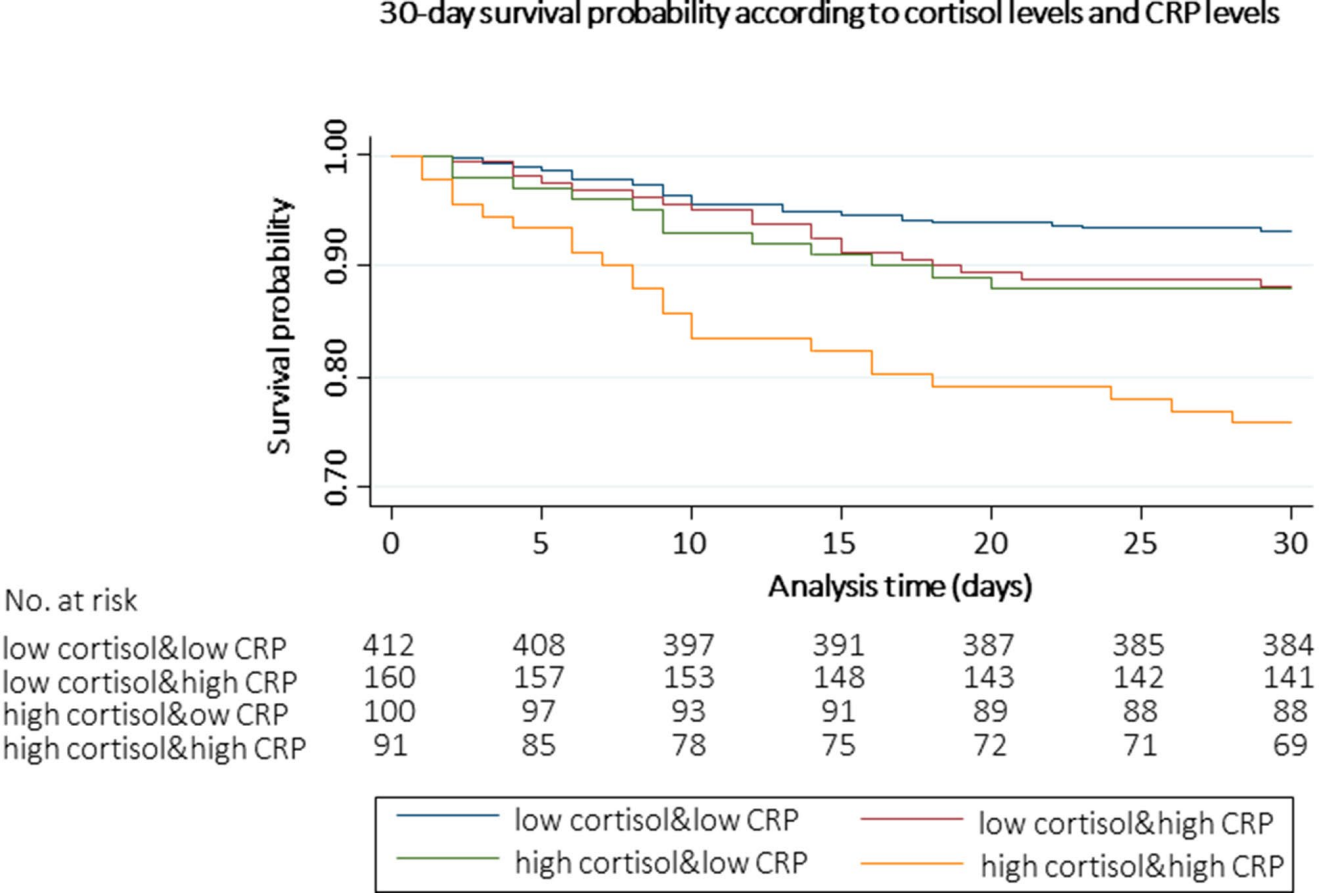
Survival probability over 30 days stratified by cortisol quartiles and CRP levels Abbreviation: CRP, C-reactive protein; No, number adjusted for age, sex, NRS, main diagnosis, comorbidities and trial intervention low cortisol: cortisol ≤ 723 nmol/l, high cortisol: > 723 nmol/l low CRP: CRP < 100 mg/l, high CRP: ≥100 mg/l

Kaplan-Meier Survival Analysis (Fig. 2) illustrates the survival probability over 30 days for patients with different combinations of CRP and cortisol levels. Patients with high CRP and high cortisol levels had the greatest risk of dying within the first 30 days, while patients with low CRP and low cortisol levels had the lowest risk.

### Effect of nutritional support on primary and secondary endpoints, stratified by cortisol levels

**Fig. 3:**
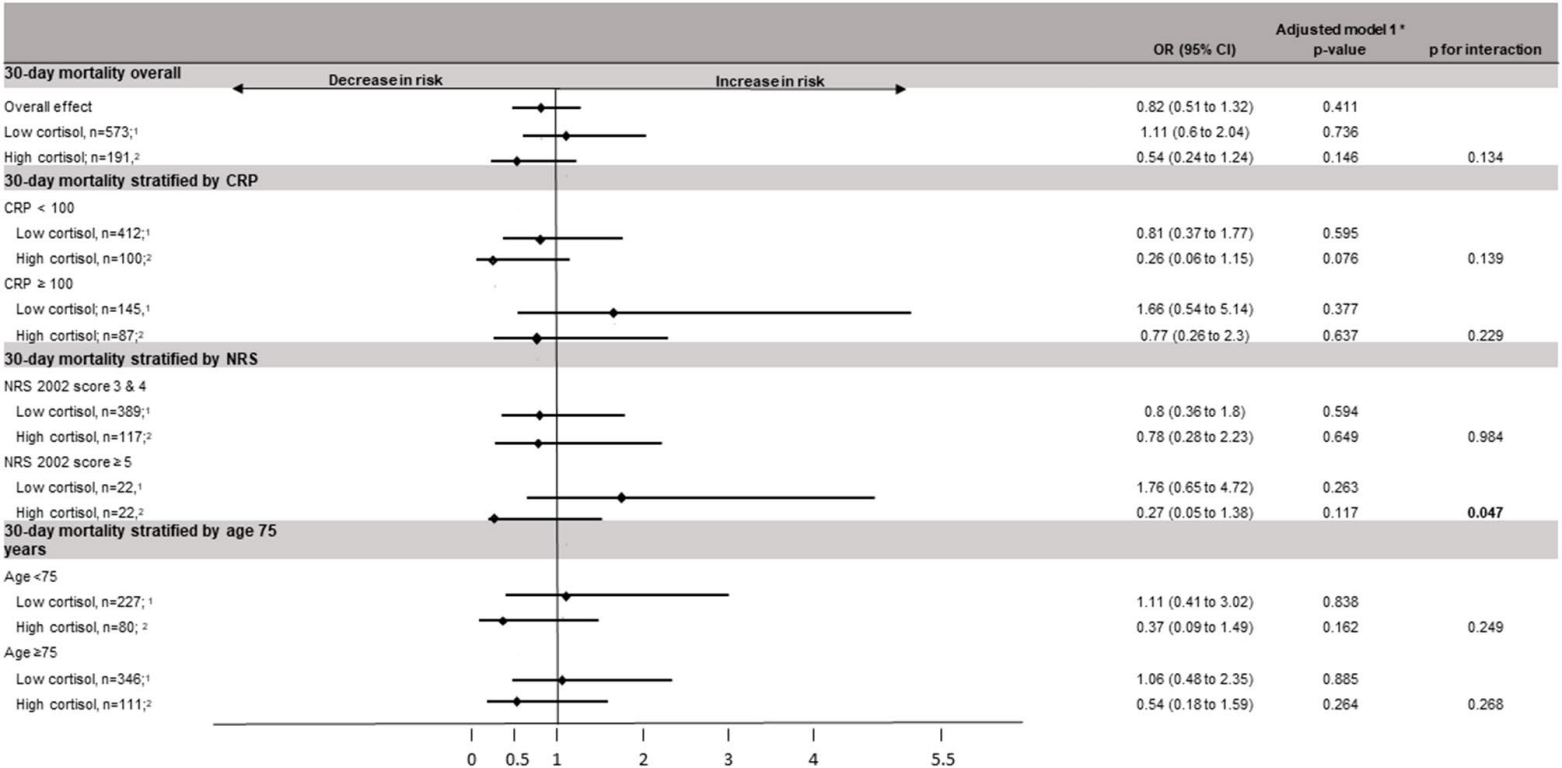
Association of nutritional support and 30-day mortality overall, stratified by cortisol levels and divided into various subgroups Abbreviations: OR, odds ratio; 95% CI, 95% confidence interval; NRS, nutritional risk scale 2002 score; CRP, C-reactive protein * adjusted for age, sex, NRS, main diagnosis and comorbidities ^1^ Low cortisol: ≤ 723 nmol/l ^2^ High cortisol: > 723 nmol/l

Finally, we evaluated the impact of nutritional support on mortality stratified by cortisol quartiles (Fig. 3). When compared to patients with low cortisol levels, those with high results showed a more pronounced therapeutic effect of nutritional intervention on 30-day mortality (adjusted OR 0.54, [95% CI 0.24 to 1.24], p = 0.146 vs. adjusted OR 1.11, [95% CI 0.6 to 2.04], p = 0.736, respectively). The interaction analysis, however, did not reach the level of significance (p for interaction 0.134). In one subgroup analysis, we found a more pronounced treatment response in severely malnourished patients with high cortisol levels (OR 0.27, [95% CI 0.05 to 1.38], p = 0.117 vs. OR 1.76, [95% CI 0.65 to 4.72], p = 0.263; p for interaction 0.047). However, when adjusting for multiple testing, this interaction is no longer significant.

## Discussion

We can draw three main conclusions from this secondary analysis of a randomized nutritional trial. First, patients with higher risk of malnutrition, reduced food intake, and appetite loss show significantly increased cortisol levels. Second, cortisol levels at time of admission to hospital have strong prognostic implications in terms of short- and long-term mortality and other clinically relevant outcomes. And third, the effect of nutritional treatment on mortality tends to be more pronounced in patients with high cortisol levels compared to patients with lower cortisol levels; especially in the severely malnourished population.

Pathophysiologically, the association of cortisol levels and nutritional parameters is of high interest. In our cohort, patients experiencing loss of appetite and reduction of food intake had higher cortisol levels compared to patients without appetite impairment. Conversely, the appetite-increasing effect of cortisone treatment or hypercortisolism induced by Cushing’s disease per se is well known [18]. Importantly, in the setting of acute illness leading to hospitalization, other factors such as proinflammatory cytokines (TNF-a, IL-1 and IL-6) and reduction of other anabolic pituitary hormones may have a stronger impact on appetite regulation [19, 20]. As cortisol is a pluripotent stress hormone that acts on different tissues to regulate pleiotropic aspects of metabolism in favour of energy provision for critical organs, one mechanism might also counteract loss of appetite and prevent further catabolism in patients with malnutrition. These effects are comparable to Cushing’s syndrome, where corticosteroid excess leads to severe morbidity and increased mortality [4, 18]. Although cortisol increases appetite via stimulation of the central nervous system, the appetite-reducing effect of cytokines most likely dominates in the face of acute illness [2, 21–23]. Chronic diseases especially delay and suppress anabolic processes and lead to persistent cachexia [20, 24]. This could explain why higher cortisol levels do not increase appetite in patients with malnutrition risk.

High cortisol levels had a strong prognostic value regarding mortality and adverse outcomes in our cohort of patients at nutritional risk. This result is in line with findings of previous studies which have described associations between cortisol levels and poor prognosis in other cohorts, including acute coronary syndrome [25], sepsis [15], and Covid-19 infection [10]. This association may be explained by the higher disease severity of patients with increased cortisol levels; alternatively, the catabolic effects of cortisol may also have deleterious effects on outcomes. As cortisol induces glycogen, fat, and protein breakdown, anabolic processes are delayed and suppressed when faced with prolonged stress - resulting in a breakdown of muscle tissue and loss of lean body mass, as usually seen in patients with Cushing’s Syndrome [18].

Evidence of the positive effects of nutritional therapy on clinical outcomes in the medical inpatient population has been growing in recent years [16, 26, 27]. However, previous analyses form our cohort and other studies suggested that not all patients benefit from nutritional intervention in the same way [17, 28–32]. This opens the door for more personalized nutritional medicine focusing on patients with most expected benefit from treatment [33]. Specific biomarkers reflecting pathophysiological pathways including specific proteins (e.g., albumin, prealbumin [34, 35]) and endocrine markers (e.g. thyroid hormones [32]) may play a particularly important role here due to their involvement in the pathophysilogy of malnutrition [33]. The current analysis suggests a pronounced effect of nutritional treatment in regard to mortality reduction in patients with high admission cortisol levels. One may therefore hypothesise that once adequate individualized nutritional therapy is offered, the impact of cortisol shifts in favour of anabolic effects, and patients with high cortisol may benefit more from nutritional therapy. As overeating can lead to reduced autophagy (a mechanism needed to dispose of cellular waste), this could account for the cytokine-induced reduction in food intake in acute disease, which in turn counteracts the slowing of cellular waste disposal [36, 37]. In a previous analysis, we found patients with high inflammation and a CRP level above 100 mg/l to show less benefit from individualized nutritional therapy compared to patients with levels lower than 100 mg/l [17]. The study hypothesized that inflammation - rather than infection - might be causing the lack of response to nutritional therapy. This would be consistent with our current findings, as patients with low CRP levels and high cortisol levels had more benefit from nutritional therapy compared to those with high CRP and high cortisol levels. According to our study, patients with high cortisol and an NRS of 5 or more had most benefit from nutritional therapy; with significant effect modification even after adjustment. Nevertheless, the prophylactic administration of cortisol in critically ill patients should be avoided as the side effects of cortisol therapy may predominate in these cases [4].

### Strengths and limitations

Following an intensive search, we believe this secondary analysis of a randomized controlled clinical trial to be the first to investigate the predictive value of cortisol levels in a population of malnourished medical inpatients. We also performed analyses which stratified for CRP levels to investigate the effect of inflammation. There are, however, several limitations to this study. First of all, the sample size may have been too small to produce significant interactions in some of the outcomes. There is also a possible selection bias due to some missing cortisol test results. Although the circadian rhythm of cortisol was not investigated, most measurements were taken in the morning and from acutely ill patients - where circadian rhythms are disturbed regardless. We did not record the actual time of blood draw and were thus not able to adjust the analysis accordingly. Due to the undefined cut-off for high cortisol, we choose quartiles which could be difficult to compare with future studies. Finally, as this is a secondary analysis, our results are hypothesis-generating rather than definitive; and require validation in an independent sample.

## Conclusion

This secondary analysis of a randomized nutritional trial suggests that cortisol levels are linked to nutritional and clinical outcome among multimorbid medical patients at nutritional risk and may help to improve risk assessment, as well as response to nutritional treatment.

### Electronic supplementary material

Below is the link to the electronic supplementary material.

Supplementary Material 1

## Data Availability

Data will be made available to others with the publication of this manuscript, as already outlined in the primary EFFORT publication, on receipt of a letter of intention detailing the study hypothesis and statistical analysis plan. A signed data access agreement is required from all applicants. Please send requests to the principal investigator of this trial.
