# Supplementary material for: Association of admission cortisol levels with outcomes and treatment response in patients at nutritional risk: A secondary analysis of a randomized clinical trial
Source: Nutr J. 2023 Nov 15;22:59. doi: 10.1186/s12937-023-00881-6 (PMC10648344; doi:10.1186/s12937-023-00881-6)
Supplement: Supplementary file 1 — Supplementary Material 1 [file 12937_2023_881_MOESM1_ESM.docx]

**Supplemental Material**

**Association of admission cortisol levels with outcomes and treatment response in malnutrition**

**A Secondary Analysis of a Randomized Clinical Trial**

Short Title: The role of cortisol in malnourished medical inpatients

Mirsada Durmisi^1,2*^, Nina Kaegi-Braun^1*^, Natasha A. Müller^1,2^, Carla Gressies^1^, Pascal Tribolet ^1,3,4^, Zeno Stanga ^5^, Beat Mueller ^1,2^, and Prof Philipp Schuetz^1,2^ for the EFFORT study team

^*^ Equally contributed shared first authorship of MD and NK-B

^1^ Medical University Department, Division of General Internal and Emergency Medicine, Kantonsspital Aarau, Tellstrasse 25, CH-5001 Aarau, Switzerland; ^2^ Medical Faculty of the University of Basel, Basel, Switzerland; ^3^ Department of Health Professions, Bern University of Applied Sciences, Bern, Switzerland; ^4^ Faculty of Life Sciences University of Vienna, Vienna, Austria; ^5^ Division of Diabetes, Endocrinology, Nutritional Medicine, and Metabolism, Inselspital Bern, Bern University Hospital, University of Bern, Switzerland

**Correspondence and reprint requests:**

Prof. Dr. med. Philipp Schuetz, MD, MPH

University Department of Medicine

Kantonsspital Aarau

Tellstrasse 25

CH-5001 Aarau, Switzerland

Email: [schuetzph@gmail.com](mailto:schuetzph@gmail.com)

**List of initial trial Steering Committee members:**

Philipp Schuetz, Filomena Gomes, Rebecca Fehr, Claus Hoess, Vojtech Pavlicek, Christoph Henzen, Jacques Donzé, Zeno Stanga, Beat Mueller

**Full list of local investigators of initial trial:**

- Kantonsspital Aarau: Thomas Baumgartner; Valerie Bächli; Luca Bernasconi; Lisa Bonoure; Manuela Deiss; Andreas Eckart; Rebecca Fehr, Susan Felder; Natalie Friedli; Martina Geiser, Filomena Gomes, Lena Grädel; Andreas Huber; Daniel Koch; Alexander Kutz; Svenja Laukemann; Olivia Neeser; Manuela Nickler; Marc Meier; Marc Meili; Beat Mueller; Jonas Odermatt; Manuel Ottiger; Isabel Pulvermüller; Anna Christina Rast; Katharina Regez; Ramon Sager; Diana Sbiti; Ursula Schild; Philipp Schuetz; Deborah Steiner; Alaadin Vögeli; Yannick Wirz; Esther Wyrsch; Giedre Zurauskaite; Seline Zurfluh;
- Inselspital Bern: Evelyne Abgottspon; Drahomir Aujesky; Adrian Baumann; Kathrin Blaser; Jacques Donzé, Tanja Flückiger; Cindy Groen; Daniela Häfeli; Corinne Hänzi; Katrin Lengacher; Melisa Merdanovic; Sarah Pfaffen; Nicolas Rodondi; Nathalie Schwab; Zeno Stanga; Monica von Brevern; Sophie von Lerber;
- Spital Lachen: Thomas Bregenzer; Bruno Schiesser; Pascal Tribolet; Anita Wild;
- Luzerner Kantonsspital: Daniela Buhl, Christoph Henzen; Silvia Mattmann; Melina Nigg; Sara Ramseier; Michael Trummler
- Kantonsspital Münsterlingen: Nina Braun; Claus Hoess, Cornelia Ebneter; Dominique Mannhart; Vojtech Pavlicek, Sarah Schmid
- Bürgerspital Solothurn: Cornelia Albrecht; Claudia Brand; Katharina Ilic; Lisa Tanner; Robert, Thomann; Rahel von Felten;
- Kantonsspital St.Gallen: Carmen Benz; Michael Brändle, Stefan Bilz; Madlaina Höhener; Sarah Sigrist; Rahel Stadler; Alexandra Wick
- Kantonsspital Baselland, Standort Bruderholz: Jonas Rutishauser; Marianne Waldmeier

**Definition of outcomes assessed during the initial trial:**

- The primary composite endpoint consists of adverse clinical outcomes within 30 days defined as follows:
  1. Mortality: all-cause mortality from inclusion to day 30
  2. ICU admission: admission to the intensive care unit from the medical ward from inclusion to day 30
  3. Non-elective hospital readmission: non-scheduled hospital readmission after discharge from the index hospital stay to day 30
  4. Major complications: any of the following complications occurring between inclusion (i.e., not present at the time of inclusion) and day 30
     1. Adjudicated nosocomial infection or abscess requiring antibiotic treatment
     2. respiratory failure requiring invasive or non-invasive ventilation (continuous positive airway pressure, CPAP)
     3. major cardiovascular events including stroke, intracranial bleeding, cardiac arrest, myocardial infarction (with and without invasive procedure), and pulmonary embolism
     4. acute renal failure (2x increase of baseline creatinine or new requirement for dialysis due to volume overload or electrolyte disturbance)
     5. gastro-intestinal events (hemorrhage, intestinal perforation, pancreatitis [minimum 2 out of 3 criteria: abdominal pain, 3-fold increase in lipase or pancreas-specific amylase, characteristic imaging findings])
  5. decline in functional status of 10% or more from admission to day 30 measured by the Barthel`s index,^1^ which measures performance in activities of daily living and comprises two categories: one related to self-care (feeding, grooming, bathing, dressing, bowel and bladder care, and toilet use), the other related to mobility (ambulation, transfers, and stair climbing). We used the German translation which has scores ranging from 100 to 0, with lower numbers indicating more severe disability.
- Secondary endpoints assessed were defined as follows:

1. every component of the primary endpoint at day 30
2. daily protein and energy intake as assessed by clinical nurses and trained registered dieticians recording each patient’s meal
3. length of hospital stay: total inpatient days during the index hospital stay from inclusion to day 30
4. quality of life measured on admission and at 30 days via the EuroQol Group 5-Dimension Self-Report Questionnaire.^2^ This included the European Quality of Life 5 Dimensions index (values range from 0 to 1, with higher scores indicating better life quality) and the visual-analogue scale (EQ-5D VAS) (scores range from 0 to 100, with higher scores indicating better health status).

- Safety endpoints including side effects from nutritional therapy are assessed daily until hospital discharge and are defined as:
  1. adverse gastrointestinal effects: obstipation, diarrhea, nausea, vomiting, abdominal pain
  2. complications related to enteral nutrition (tube feeding) or parenteral nutrition (any complications associated with central venous catheter)
  3. refeeding syndrome: according to a recent consensus definition^3,4^ as electrolyte shifts (e.g., decrease in Phosphate, Magnesium, Potassium) suggestive of refeeding syndrome in conjunction with typical clinical symptoms (e.g., peripheral or acute lung edema)
  4. liver or gall bladder dysfunction
  5. hyperglycemia (persistent levels >10mmol/l in patients without diabetes or well controlled diabetes)

**Supplemental Tables**

**References**

1. Mahoney FI, Barthel DW. Functional Evaluation: The Barthel Index. Md State Med J 1965;14:61-5.

2. Brooks R. EuroQol: the current state of play. Health Policy 1996;37:53-72.

3. Friedli N, Stanga Z, Sobotka L, et al. Revisiting the refeeding syndrome: Results of a systematic review. Nutrition 2017;35:151-60.

4. Friedli N, Stanga Z, Culkin A, et al. Management and prevention of refeeding syndrome in medical inpatients: An evidence-based and consensus-supported algorithm. Nutrition 2018;47:13-20.
